# Ethnobotany, Phytochemistry and Pharmacological Activity of *Kigelia africana* (Lam.) Benth. (Bignoniaceae)

**DOI:** 10.3390/plants9060753

**Published:** 2020-06-15

**Authors:** Alice Nabatanzi, Sanah M. Nkadimeng, Namrita Lall, John D. Kabasa, Lyndy J. McGaw

**Affiliations:** 1 Department of Plant Sciences, Microbiology and Biotechnology, College of Natural Sciences, Makerere University, Kampala 00256, Uganda; 2 Phytomedicine Programme, Department of Paraclinical Sciences, Faculty of Veterinary Science, University of Pretoria, Onderstepoort 0110, South Africa; sanah.nkadimeng@up.ac.za (S.M.N.); lyndy.mcgaw@up.ac.za (L.J.M.); 3 Department of Plant and Soil Sciences, University of Pretoria, Hatfield 0028, South Africa; namrita.lall@up.ac.za; 4 College of Veterinary Medicine, Animal Resources and Biosecurity, Makerere University, Kampala 00256, Uganda; kabasa@covab.mak.ac.ug; 5 Future Africa, University of Pretoria, Hatfield 0028, South Africa; 6 School of Natural Resources, University of Missouri, Columbia, MO 65211, USA; 7 College of Pharmacy, JSS Academy of Higher Education and Research, Mysuru, Karnataka 570015, India

**Keywords:** *Kigelia africana*, phytochemistry, traditional uses, pharmacological activity, Bignoniaceae

## Abstract

*Kigelia africana* has been used in the management of human ailments since time immemorial. Ethnobotanists have documented the traditional uses of *K. africana*, which include treatment of skin disorders, cancer and gynecological complaints, among others. This has interested scientists, who have examined *K. africana* plant parts for their bioactivity. This review provides an insightful understanding on the ethnobotany, phytochemistry and pharmacology of *K. africana*. Web search engines Google and Google Scholar, as well as the databases of PubMed, Scopus, JSTOR, HINARI, SID, AJOL and Springer Link, were exhaustively searched using key words and phrases. Institutional reports and conference papers were also consulted. A total of 125 relevant international literature sources meeting the inclusion criteria were included. *Kigelia africana* has biologically active phytochemicals, many of which have been isolated. Whilst the fruits are most often cited in pharmacological studies, other plant parts are also used in herbal preparations. Commercially available products have been formulated from *K. africana,* though many have not been fully standardized. Despite many efforts by researchers to scientifically validate traditional uses of *K. africana*, many remain merely claims, thus the need to conduct more research, scientifically validate other traditional uses, isolate new bioactive phytochemicals and standardize *K. africana* products.

## 1. Introduction

Traditional medicine (TM) plays a very significant role in indigenous health care systems for humans, especially in developing countries where access to allopathic medicines and practitioners is limited. Throughout Africa, communities have relied on TM for centuries, because it is easy to access, is culturally appropriate and is considered safe. The practice of TM includes explicit practices that exploit materials from plants, animals and inorganic materials (e.g., soils) and implicit methods that include libations to spirits. Plant materials from medicinal plants find the widest application in TM preparations [1]. In turn, this has threatened the plant species from which such materials are sourced. Furthermore, most of the plant resources are threatened by unsustainable harvesting, habitat modification and conversion [2]. *Kigelia africana* (Lam.) Benth. is one of those tree species that have been heavily exploited for their medicinal, religious and cultural values [3]. It belongs to family Bignoniaceae and is the only species in the genus *Kigelia*. Bignoniaceae is the trumpet creeper or catalpa family of the mint order of flowering plants (Lamiales). It contains about 110 genera and more than 800 species of trees, shrubs and, most commonly, vines, chiefly of tropical America, tropical Africa and the Indo-Malayan region. *Kigelia africana* is endemic to Africa and distributed throughout the continent but does not occur in Mauritania, São Tomé and Principe and the Indian Ocean Islands [4,5]. *Kigelia africana* has several synonyms, some of which include: *Kigelia pinnata* (Jacq.) DC., *Bignonia africana* Lam., *Crescentia pinnata* Jacq., *Kigelia abyssinica* A. Rich., *Kigelia aethiopica* Decne., *Kigelia aethiopum* (Fenzl) Dandy, *Kigelia erytraeae* Mattei, *Kigelia ikbaliae* De Wild., *Kigelia somalensis* Mattei, *Kigelia acutifolia* Engl. ex Sprague, *Kigelia elliotii* Sprague, *Kigelia elliptica* Sprague, *Kigelia impressa* Sprague, *Kigelia spragueana* Wernham, *Kigelia talbotii* Hutch. & Dalziel, *Tanaecium pinnatum* (Jacq.) Willd. and *Tecoma africana* (Lam.) G. Don). The common name of *K. africana* is “sausage tree”, which is derived from the shape of its fruits, which look like a sausage. This tree species grows mostly in the wild in wet areas along watercourses, in riverine fringes, alluvial and open woodlands, high-rainfall savannas, shrublands and in rain forests [6,7]. It occurs on loamy red clay soils, sometimes rocky, damp or peaty, from sea level up to 3000-m altitudes. Although local communities tend to protect *K. africana* saplings while in the wild, enabling them grow to maturity, this tree is sometimes domesticated and most often propagated by seeds [8]. 

The local names of *K. africana* vary throughout Africa due to the ethnic and cultural diversity on the continent. The many dialects reflect *K. africana*’s ethnobotanical significance among several African communities [9]. Some of *K. africana*’s local African names include: Nufuten, Nanaberetee (Ashanti and Akwapem), Etua (Fante), Blimmo (Baule), Akpele (Ga), Lele (Adanme), Nyakpe (Ewe), Rawuya (Hausa), Jilahi (Fulani), Bulungu (Kanuri), Bechi (Nupe), Pandoro (Yoruba), Ugbongbon (Bini), Uturubein (Ibo), Abu Shutor, Abu Sidra, Um Shutur, Umm Hashatur (Arabic), Rangbarabgbo (Zande), muVeve (Tonga), muVumati (Ndau), muZunguru (KalanBa), mPolota (Lozi), umBvewe, iPfungwani, muBvee (Shona, Zezuru, Manyika), Mufungufungu (Bemba and Lozi), ~lunguli (Lozi) Muzungule (Lozi and Tonga), Kufungule (Kaonde) Ifungufungu, Mufunofuno (Lunda), Chizutu, Mvula, Mvunguti (Nyanja). Muratina, (Kikuyu and Meru), Muatini, Kiatine (Kamba), Hwasini, Mvongonia (Teita), Ol-Suguroi, Ol-Darpoi (Masai), Yago (Luo), Morabe (Kakamega), Mvungunya, Mvungavunga, Hwegea, Mwicha, Mranaa (Swahili), Muratini (Gitiama), Mukisha (Taveta), Ratiunet (Nandi), Ratiunet (Kipsigi), Sheole (Boni), Modukguhlu (Sepedi), Muvevha (Venda), Worsboom (Afrikaans), Hantsar giiwaa (Hausa), Yago (Acholi), Edodoi (Ateso), Sifungu (Lugisu), Naizungwe (Lusoga), Omusa (Luganda), Roti (Pokot) and Bukuraal (Somalia). The many traditional medicinal uses of *K. africana* have attracted significant scientific interest in the species’ pharmacological activity. 

Although many in vitro efficacy studies of *K. africana* have been reported by several researchers, more work needs to be done to clinically prove *K. africana*’s efficacy in vivo before clinical applications. This paper summarizes the current information on the traditional uses and pharmacological and commercial significances of *K. africana*. 

## 2. Methods

Databases of PubMed, Scopus, JSTOR, Mendeley, Scientific Information Database (SID), HINARI, Springer Link and African Journals OnLine (AJOL) were searched, in addition to Google Scholar. Institutional reports, student theses, educational newspaper articles, educational magazines and conference papers were also consulted. “*Kigelia africana*”, “*Kigelia pinnata*”, “phytochemicals”, “traditional uses”, “*K. africana* products”, “biological activity”, “pharmacological activity” and “Africa” were the keywords used in the web search engines. Nonetheless, other phrases reflecting subjects of interest were used. During the search process, the keywords were used in a variety of combinations, which included synonyms, alternative terminologies, alternative spelling, related terms and variations in word endings. Boolean operators, viz., “AND”, “OR”, “-”, “NOT” and “+” were used to combine and exclude terms when searching within Google and Google Scholar. Wildcard operators, viz., (*), (?), (~) and (!) were used when searching the databases. Examples of topics explored include: traditional medicine, indigenous knowledge, medicinal plants, antifungal activity, antibacterial activity, antiplasmodial activity, analgesic activity, anti-inflammatory activity, antioxidants, anticancer activity, toxicity, commercial products, *Kigelia africana*, botany and ethnobotany, among others. The search was conducted over a five-month period and included references published up until January 2020. All publications resulting from these searches were screened, and pertinent records were collated using Mendeley, a web-based bibliography database manager. Both in vitro and in vivo studies were included. All documents that met the inclusion criteria of the review paper were retrieved and evaluated by the authors. While the authors strove to identify all pertinent documents, some literature may have been inadvertently omitted, such as unpublished student theses, conference proceedings and grey literature. Inclusion criteria for this review included: (1) English-language documents, (2) published primary and secondary literatures, (3) published student theses, (4) newspaper articles and (5) technical reports. While *K. africana*’s significance in the traditional indigenous system of Africa was the primarily focus of the review, studies carried out on *K. africana* outside Africa were also included. 

## 3. Results and Discussion

An overview of the four topic areas that are presented in this section: (1) botanical description of *K. africana*, (2) ethnobotany, (3) phytochemistry of *K. africana* and (4) pharmacological activity of *K. africana.*

### 3.1. Botanical Description 

*Kigelia africana* (Lam.) Benth. syn. *Kigelia pinnata* belongs to the family Bignoniaceae and is the only species in the genus *Kigelia* [10]. The generic name *Kigelia* comes from the Mozambican name for sausage tree, “*kigeli-keia”*. *Kigelia africana* is native to Africa, thus the derivation of the species name “*africana*”. The tree is deciduous, with a rounded crown, thick trunk, dark-grey to light-brown, scaly slash creamy-white with a green edge, low-branching, branches and branchlets spreading and lenticellate [11]. The tree reaches maturity within four to six years, with a height of up to 24 meters [11]. The leaves are alternate, pinnate and stipules absent; rachis up to 50 cm long; leaflets three to six opposite pairs, usually with a terminal leaflet, elliptic to elongated lanceolate, 7–20 cm long, 4–12 cm wide, apex abruptly to gradually shortly acuminate, base slightly asymmetrical, rounded to cuneate, margins entire or sometimes slightly toothed, coriaceous or papyraceous, shiny green and usually scab rid above, dull green and glabrous to tomentose below; midrib impressed above, major lateral veins 7–12 pairs and prominent below [11]. The flowers of *K. africana* are hermaphrodite, zygomorphic and five-merous. The calyx is campanulate approximately 1–4-cm-long, 1–2-cm-wide, fleshy, irregularly five-lobed, the lower lobes generally longer at maturity and the calyx mouth thus oblique. The corolla is greenish-yellow to purplish-red or bright claret, 5–12-cm-long, the throat rather abruptly expanded, limb 9–18 across with the two upper lobes smaller than the three lower and velvety inside; stamens four fertile and one staminode about half the fertile stamens. The ovary is conical, tapering into a slender style subequalling the stamens [12,13,14,15,16]. They possess a very unpleasant scent, which is most notable at night, indicating their reliance on pollination by bats, which visit them for pollen and nectar [16]. The fruits are indehiscent, woody, greyish-brown, sausage-shaped and pendulous, up to 50-cm-long and 15 cm in diameter, with elongated pedicels. The seeds are numerous, unwinged, obovate and 1.25-cm-long [12,13,14,15]. The fruits usually weigh 10 kg [4]. The mature fruits can be found on trees throughout the year [6,16]. Although not eaten by humans, they find wide applications in traditional medicine [11]. Due to the unusual fruits and large attractive flowers, *K. africana* is considered a striking ornamental plant, and the fruits are used as florists’ materials. The thick stem is an attractive feature for bonsai. The tree is sometimes planted as a boundary marker but usually at roadsides and for shade. Due to its occurrence along watercourses, it is suitable for erosion control and riverbank stabilization. 

### 3.2. Ethnobotany

Plant species in the Bignoniaceae family play a central role in traditional medicine systems [17], and *K. africana* is no exception. The preparation and use of *K. africana* plant parts in traditional medicine differs across and within communities. Despite the differences in preparation and application, there is still a lot of overlap, viz., similar uses in different regions or countries. *Kigelia africana* has interested many ethnobotanists and cultural anthropologists across the world who have intensively engaged in documenting its uses in several communities. The fruits are used in ethnoveterinary medicine to treat digestive system disorders, leg edemas, dermal irritations and infections, mastitis, retained placenta, brucellosis and Newcastle disease. The tree provides a nutritious food source during times of famine: the hard seeds are roasted and eaten. The fruit pulp, however, is said to be inedible and toxic, may have intoxicant or purgative effects and may cause blistering of the tongue and skin. However, fallen fruits, along with leaves and flowers, are browsed or foraged by livestock and game [10]. Fruits and bark are in the brewing process to aid fermentation and enhance the flavor of traditional beers. *Kigelia africana* wood is considered excellent for dugout canoes, planks and fenceposts. It is also used for making boxes, drums, stools, yokes, tool handles, mortars and large bowls for watering cattle. Weapon bows are made from branches, and smaller branches are hollowed to administer enemas to children [15]. Wood and fruits are carved into mousetraps, dolls and various items of crockery and cutlery. The wood is used as fuel. A black dye is obtained from the tannin-rich fruit pulp. *Kigelia africana* is regarded as sacred in many regions [10], and the flowers and fruits are regarded as a fetish. Fruits are commonly sold in markets as charms to promote wealth and prosperity, to impart strength and courage on warriors prior to, to increase crop yields and as a fetish for fecundity or to avert whirlwinds. The fruits and bark of *K. africana* are collected and traded locally in marketplaces.

Fruits are the most frequently used plant part in traditional medicine preparations, followed by the stem bark, roots and leaves [18]. Flowers are quite infamous and rarely used as medicine, because they are seasonal, and when they bloom, within 14 days, they fall off the tree and dry. Thus, seasonality affects their wide application in traditional medicine preparations, coupled with the short life cycle. The fruits are never consumed fresh, because they are said to be very poisonous, especially when young [19]. Whereas *K. africana* is traditionally considered potent, its pharmacological activity cannot be attributed to it as a single species, in some cases. Table 1 shows that some traditional medicine preparations involve using *K. africana* in combination with other medicinal plants or mollusks, like snails, or other foods, like porridge. This implies that these factors should be taken into consideration when examining its pharmacological activity. Unfortunately, the traditional uses of *K. africana* have threatened its existence on the African continent. The traditional uses of *K. africana* in different African communities, regions and countries, together with the plant part used, are summarized in Table 1. 

### 3.3. Phytochemistry

Whereas several compounds have been identified from *K. africana,* more studies are required to fully characterize its phytochemistry [45]. According to Table 2, iridoids and quinones have been identified in all plant parts. The stem bark has a higher diversity of phytochemicals compared to other plant parts. Alkanes are common in the leaves. Table 2 shows that not much has been done to identify phytochemicals in *Kigelia* flowers, which relates to their low degree of usage in traditional medicine preparations. *Kigelia* flowers are very attractive, thus finding wider applications as ornamentals. Monoterpenoid naphthoquinones (pinnatal, isopinnatal, kigelinol and isokigelinol) are unique to *K. africana.* Table 2 shows the classes of phytochemicals in *Kigelia africana*, the phytochemicals therein and the plant parts in which the respective phytochemicals are found.

### 3.4. Pharmacological Activity of Kigelia africana 

Many researchers who have investigated the pharmacological activity of *K. africana* have relied on a known traditional use or ethnobotanical application [12]. On the other hand, some pharmacological uses have been serendipitously discovered in laboratories. While many traditional uses have not been substantiated in the laboratory, quite a number have proven positive through comprehensive clinical trials. In turn, products or drug leads have been discovered.

#### 3.4.1. Antibacterial and Antifungal Activity

Roasted seeds, bark and fruit extracts of *K. africana* are traditionally used to treat fungal and bacterial infections (Table 1). Of the three plant parts, fruit extracts have found the widest applications in the treatment of fungal and bacterial skin infections. This may justify their wider usage in skin care formulations. In clinical microbiology, bacterial and fungal susceptibility tests are of paramount importance, as they help detect possible efficacy or resistance of common pathogens to the drug being tested [65]. Therefore, results of susceptibility tests are not an end and should always be followed by in vivo studies. This review reported susceptibility tests by Hussain et al. [16] and Arkhipov et al. [45]. 

The in vitro antibacterial activity of *K. africana* ethanolic, n-hexane and aqueous leaf, fruit and bark extracts against *Staphylococcus aureus, Proteus vulgaris, Escherichia coli, Pseudomonas aeruginosa, Klebsiella pneumoniae* and *Citrobacter amalonaticus* using the agar disc diffusion method has been investigated [16]. The experimental controls were oxytetracycline (positive control), n-hexane and ethanol (negative controls). The ethanolic leaf extract showed maximum activity against *E. coli* (22 mm) and moderate activity against *P. vulgaris* and *P. aeruginosa*, respectively. *Klebsiella pneumoniae* and *C. amalonaticus* showed resistance with no inhibition zones. For the aqueous leaf extract, maximum antibacterial activity was observed against *C. amalonaticus* (7 mm) and moderate activity against *S. aureus* (5 mm), *P. aeruginosa* (4 mm) and *P. vulgaris* (4 mm). *Klebsiella pneumoniae* showed resistance to the aqueous leaf extract, with no inhibition zone. Oxytetracycline showed a significant zone of inhibition against *S. aureus* (20 mm) and least activity against *K. pneumoniae*, while moderate values were observed against the rest of the bacterial strains. 

The n-hexane leaf extract of *K. africana* exhibited maximum activity against *P. vulgaris* (6 mm), with minimum activity against *S. aureus* (2 mm). *K. pneumoniae*, *P. aeruginosa*, *C. amalonaticus* and *E. coli* showed resistance with no inhibition zones. The aqueous *K. africana* fruit extract had the highest inhibition against *P. vulgaris* (6 mm) and *C. amalonaticus*. It showed moderate inhibition of *S. aureus* and *E. coli*. *Klebsiella pneumoniae* and *P. aeruginosa* were resistant, with no zone of inhibition. With oxytetracycline, the largest zone of inhibition was observed against *E. coli* and *P. vulgaris* (23 mm) and minimum against *C. amalonaticus*. Zones produced by the fruit of *K. africana* against all bacterial strains were highly significant. 

The bark extract of *K. africana* prepared with different solvents possessed good antibacterial activity against *E. coli* and minimum antibacterial activity against *K. pneumoniae*. The ethanol extract of *K. africana* bark had good antibacterial activity against *E. coli* (10 mm), moderate activity against *P. aeruginosa* and *P. vulgaris* and the lowest activity against *S. aureus* (1 mm), while *K. pneumoniae* and *C. amalonaticus* were resistant, with no zones of inhibition. With the n-hexane extract, *K. africana* bark produced resistance against all bacterial strains except *C. amalonaticus* (4 mm) and *E. coli* (2 mm). The aqueous bark extract of *K. africana* showed a maximum zone of inhibition against *S. aureus* (15 mm). A small zone of inhibition (5 mm) was observed against *P. aeruginosa* and *P. vulgaris*. The smallest inhibition zone was formed against *C. amalonaticus,* while *K. pneumoniae* and *E. coli* were resistant. Oxytetracycline showed the largest zone of inhibition in the cases of *S. aureus* (25 mm) and *E. coli* (20 mm). The smallest zone of inhibition was observed against *C. amalonaticus* (3 mm). Ethanolic and aqueous extracts of bark and leaves of *K. africana* showed remarkable activity against the bacterial strains as compared to n-hexane. *S. aureus* and *E. coli* were proved as highly sensitive strains, while *K. pneumoniae* was the resistant strain, as the extracts formed no inhibition zone against it.

Hussain et al. [16] employed the agar disc diffusion method, which has its own limitations. First, some species of bacteria cannot be tested accurately using this method. This implies that, before a researcher chooses which method to use for antibacterial susceptibility assays, they should be well-conversant with the class of bacteria they are dealing with. These may either be fastidious or nonfastidious bacteria. According to The European Committee on Antimicrobial Susceptibility Testing (EUCAST), fastidious bacteria will only grow well if special nutrients are present in the culture medium, viz., BD Mueller Hinton Fastidious Agar. Fastidious bacteria include *Streptococci* spp., *Neisseria gonorrhoeae*, *Haemophilus influenzae*, *Moraxella catarrhalis*, *Listeria monocytogenes*, *Pasteurella* spp., *Kingella kingae*, *Aerococcus* spp., *Campylobacter* spp., *Legionella* spp., *Brucella* spp., *Francisella tularensis*, *Leptospira* spp., *Borrelia burgdorferi*, *Bartonella* spp. and *Bordetella* spp. to mention but a few. Nonfastidious bacteria can grow without special nutritional supplements on agar plates. These include *Bacillus* spp., *Staphylococcus* spp., *Escherichia coli*, *Pseudomonas* spp., *Klebsiella* spp. and *Citrobacter* spp. Therefore, whereas the same minimum inhibitory concentration (MIC) method may be applied, slight modifications, especially in culture media, are a must when dealing with fastidious bacteria.

In another study, Arkhipov et al. [45] investigated the antibacterial and antifungal activities of *K. africana* fruit powder against 19 bacterial species, viz., *Acintobacter bayleii*, *Aeromonas hydrophila, Alcaligenes feacalis, Bacillus cereus, Citrobacter freundii, Enterobacter aerogenes, Enterococcus faecalis, Escherichia coli, Klebsiella pneumoniae, Penicillium chrysogenum, Proteus mirabilis, Pseudomonas aeruginosa, Pseudomonas fluorescens, Salmonella newport, Serratia marcescens, Shigella sonnei, Staphylococcus aureus, Staphylococcus epidermidis* and *Streptococcus pyogenes* and three fungal species, viz., *Aspergillus niger*, *Candida albicans* and *Penicillium chrysogenum* using modified disc diffusion and MIC methods. Standard discs of ampicillin (2 µg) and nystatin (100 µg) served as positive controls for antibacterial and antifungal activity, respectively. Filter discs impregnated with 10 µL of distilled water served as negative controls. 

The methanol and water extracts displayed broad spectrum inhibitory activity against Gram-negative bacteria (*A. bayleii, A. hydrophila, A. faecalis, C. freundii, E. aerogenes, E. coli, K. pneumoniae, P. mirabilis, P. aeruginosa, P. fluorescens, S. newport, S. marcescens* and *S. sonnei*), inhibiting the growth of nine (69%) and eight (62%) of the four Gram-negative species tested, respectively. The methanol extract was generally more effective at inhibiting Gram-negative bacterial growth than was the aqueous extract. Proteus mirabilis was particularly susceptible, with zones of inhibition greater than 10 mm for the methanol, water and ethyl acetate extracts. The chloroform and hexane extracts were unable to inhibit any of the bacterial species tested. The ethyl acetate extract also displayed inhibitory activity towards the Gram-negative bacteria, albeit with a narrower specificity, inhibiting three of the four bacterial species (23%). Each inhibited the growth of three of the five species (60%). In contrast, the ethyl acetate extract inhibited only S. epidermidis (20%). It is noteworthy that, whilst growth inhibition was detected for several Gram-positive bacteria, the zones of inhibition indicated that this inhibition was not particularly strong against any of these bacteria. None of the Gram-positive bacteria were inhibited by the chloroform or hexane extracts. 

The methanol, water and ethyl acetate extracts showed broad growth inhibitory activity against the fungi tested (75%). Only *P. chrysogenum* was resistant to all the extracts. The methanol extract showed the strongest inhibition to the growth of the fungi *A. niger, C. albicans* and *P. chrysogenum,* with MIC values of 1238, 841.2 and 989.7 µg/mL, respectively. The MIC values of the aqueous extract for the fungi *A. niger*, *C. albicans* and *P. chrysogenum* were 2487, 2060 and 2768 µg/mL, respectively, while the MIC values of the ethyl acetate extract were 1463, 1278 and 1744 µg/mL for the fungi *A. niger*, *C. albicans* and *P. chrysogenum,* respectively. The methanol extract of the fruit of *K. africana* had a minimum fungicidal concentration of >1 g/mL; therefore, it was not active against *C. neoformans* [66]. The authors diluted and tested the plant extracts across a concentration range of doses 5 mg/mL to 0.1 mg/mL, which increases the reliability of the results. The iridoids specioside and verminoside in *K. africana* are responsible for its antibacterial effects [42].

Arkhipov and colleagues mention using modified disc diffusion and MIC methods to determine the antibacterial and antifungal activity [45]. Unfortunately, they do not go further to clearly state how the methods were modified and why they had to modify these methods. This leaves the reader wondering whether the modification was to improve the method efficiency or to increase susceptibility of the pathogens. Additionally, percentages of inhibition by the different species were stated, but the authors did not clearly state how significant the inhibition was, which can only be achieved by stating the statistical differences, which analyses were not performed. Furthermore, the authors frequently used terms like “majority”, “narrower specificity” and “strong”. These terms are difficult to interpret, as they were not accompanied by statistics in terms of significant differences among species and in comparison, with the controls across the different concentrations. This implies that a proper statistical software had to be used for analyses in order to make the results more meaningful. 

Whenever a researcher opts to use the disc diffusion method, it is of paramount importance to use the correct media, appropriate controls, proper growth requirements and test conditions, among others [67,68]. Furthermore, with the disc diffusion method, one cannot ascertain the right extract concentration-causing effect, since the amount of diffused extract cannot be accurately quantified. This implies that a researcher who chooses to use disc diffusion should confirm the results with agar or broth dilution tests, which are also able to indicate MIC values. Whereas Hussain et al. [16] and Arkhipov et al. [45] stated the bacterial strains used in the respective studies, they did not mention the strain collection numbers, which would be useful as a comparison with other studies. Strain collection numbers are recommended by the EUCAST (European Committee on Antimicrobial Susceptibility Testing) for purposes of quality control [69] throughout the experiment. The EUCAST-recommended strain distributers include: ATCC (American Type Culture Collection, USA), NCTC (National Collection of Type Cultures, UK), CIP (Collection de Institut Pasteur, France), DSM (Deutsche Stammsammlung fur Mikroorganismen und Zellkulturen, Germany), CCUG (The Culture Collection University of Gothenburg, Sweden) and CECT (Coleccion Espanola de Cultivos Tipo, Spain) [69]. Therefore, since strain collection numbers are not stated in these studies, it directly impacts on the reliability of results and reproducibility of the experiment. 

Gaps in these studies can be extrapolated to similar tests, as researchers always overlook the same. Whenever a drug or plant extract being tested has a good minimum inhibitory concentration (MIC), researchers usually conclude that the test drug has good antibacterial activity, which may not be right. This is because MIC results can only ascertain that the growth of bacteria under study is inhibited, but the pathogens may not be dead. Thus, there is a strong possibility that the bacteria will grow again if a conducive environment is provided. Additionally, it is often not known or determined whether antimicrobial activity is due to a general toxicity to all cells or whether this is a selective activity against the bacteria or fungi.

#### 3.4.2. Analgesic and Anti-Inflammatory Activity

For centuries, medicinal plants have been used to manage inflammation among several indigenous communities [70]. The roasted seeds, fruit infusion and bark of *K. africana* are traditionally used in the treatment of waist pain, back pain and inflammation (Table 1). Many conventional anti-inflammatory drugs work by inhibiting cyclooxygenase (COX), the enzyme that makes prostaglandins (PGs) [71]. However, herbal medicines act via different pathways, one of which is by inhibiting nuclear factor-kB (NF-kB) inflammatory pathways [72]. NF-kB can detect noxious stimuli, such as infectious agents, cellular injuries and free radicals, and then promotes the synthesis of inflammatory cytokines. Inhibition of NF-kB leads to the management of inflammation [73]. 

Kamau [74] investigated the anti-inflammatory activity of the fresh stem bark and leaves of *K. africana* using carrageenan-induced right paw edema in mice, as described by Winter et al. [75]. Thirty Swiss albino mice of either sex were divided randomly into six groups of five mice each and treated as follows; Group I (normal control) was not induced with paw edema but received 4% dimethylsulphoxide (DMSO). Group II (negative control) was induced with paw edema and received 4% DMSO. Group III (positive control) was induced with paw edema and received diclofenac (reference drug) at a dose of 15 mg/kg body weight. Groups IV, V and VI (experimental groups) were induced with paw edema and received the extracts at a dosage of 50 mg/kg, 100 mg/kg and 150 mg/kg body weight. Acute inflammation was induced by a subplantar injection of 0.05 mL 1% carrageenan (Sigma—type I) in normal saline 30 min after treatment. The change in paw diameter was measured using a digital Vernier caliper 30 min before the injection of carrageenan and at 1, 2, 3 and 4 h after the induction of inflammation [76]. 

The methanolic leaf extract of *K. africana* showed significant anti-inflammatory activity on carrageenan-induced paw edema, which was demonstrated by the reduction in the inflamed hind paw diameter after the extract administration. In the first hour, the leaf extract of *K. africana* at the dose of 150 mg/kg and reference drug diclofenac at the dosage of 15 mg/kg body weight showed anti-inflammatory effects by reducing the hind paw diameter by 0.21% and 1.10%, respectively. However, the extract at the dosages of 50 mg/kg and 100 mg/kg body weight showed no anti-inflammatory activity during the first hour. In the second hour, the leaf extract of *K. africana* at the doses of 100 mg/kg and 150 mg/kg body weight, as well as diclofenac (reference drug), reduced the inflamed paw diameter by 0.42%, 1.42% and 2.8%, respectively. However, the extract at the dosage of 50 mg/kg body weight did not show anti-inflammatory activity currently. The anti-inflammatory activity of the extract at the dose levels of 50 mg/kg and 100 mg/kg showed no significant differences (*p* > 0.05).

The anti-inflammatory activity of the extract at a dose of 150 mg/kg body weight was comparable to diclofenac (reference drug) (*p* > 0.05). In the third hour, the extract at the dose levels of 50 mg/kg, 100 mg/kg and 150 mg/kg body weight, as well as diclofenac, reduced the inflamed hind paw diameter by 0.86%, 2.25%, 3.41% and 4.02%, respectively. The anti-inflammatory activity of the extract at the dosages of 50, 100 and 150 mg/kg body weight showed no significant differences and were comparable to the activity of diclofenac (*p* > 0.05). In the fourth hour, the leaf extract of *K. africana* at the dose levels of 50, 100 and 150 mg/kg body weight reduced the inflamed hind paw diameter by 1.95%, 2.98% and 4.98%, respectively. Similarly, the reference drug reduced the inflamed paw diameter by 4.43% at this time. The anti-inflammatory activity of the extract at the dosages of 50 and 100 mg/kg body weight showed no significant difference (*p* > 0.05). In addition, the anti-inflammatory activity of the extract at 150 mg/kg body weight was comparable to that of diclofenac (*p* > 0.05). The concentration of diclofenac was 15 mg/kg. The anti-inflammatory activity of the methanolic leaf and stem back extracts of *K. africana* was dose-dependent after the second hour of the test period, with the dose level of 150 mg/kg body weight, producing the greatest anti-inflammatory activity. Thus, anti-inflammatory activity increased with an increase in the extract concentration, as evidenced by 150 mg/kg reducing the hind paw diameter by 4.98% in the fourth hour, which was more effective than the reference drug diclofenac. 

Namita et al. [77] investigated the analgesic properties of the *K. africana* methanolic leaf extract using Wistar rats against the standard drug pentazocine (10 mg/kg) by hot plate and the tail flick method. Wistar rats were divided into four groups of five animals each. The control group received normal saline (0.01 mL), group 2 (standard drug group) received pentazocine (10 mg/kg, i.p.), group 3 received *K. africana* methanolic leaf extract (200 mg/kg, p.o.) and group 4 received *Kigelia* methanolic leaf extract (400 mg/kg, p.o.). The extract (*p* < 0.001) prolonged the reaction time at different time intervals, and the effect was dose-dependent. The analgesia began at 60 min, remained for 120 min and the peak effect was noted at 90 min in comparison to the control. The higher dose of the extract (400 mg/kg) exhibited better analgesic activity than the lower dose at (200 mg/kg), and the standard drug pentazocine (10 mg/kg, i.p.) showed highly significant analgesic activity (*p* < 0.001) in a dose-dependent manner. At 60 min, analgesia began, remained for 120 min and peak activity was noted at 90 min in comparison to the control. Nonetheless, at extract dose of 200 mg/kg analgesia was produced at 60 min to 90 min. The standard drug pentazocine (10 mg/kg, i.p.) showed the highest significant effects. According to Arkhipov et al. [45], verminoside is responsible for the analgesic and anti-inflammatory effects of *K. africana*. Verminoside is known to cause significant anti-inflammatory effects, inhibiting both iNOS expression and NO release in the Lipopolysaccharides-induced J774.A1 macrophage cell line [60]. 

Kamau [76] showed that the methanolic leaf and stem bark extracts of *K. africana* can reduce carrageenan-induced right paw edema in mice, and extracts compare well with diclofenac (reference drug). Whereas Kamau [76] mentions using animals of either sex, the author does not state the number of male and female animals used. Therefore, one cannot tell whether the effects of the extracts are dependent on animal physiology and whether the responses to doses may differ. Furthermore, using the tail flick method to investigate the analgesic activity is quite deceptive, and the results may have a different interpretation. This is majorly because the behavioral response in the tail flick test is consistent with the tail withdrawal response as a spinal reflex rather than an indication of pain, involving higher brain centers [78]. More so, during this method, the rodent is always restrained, which may be against animal ethics if it takes a longer time. 

#### 3.4.3. Antidiabetic Activity

The use of *K. africana* fruit extracts as a treatment for diabetes is commonest among South African indigenous communities (Table 1), notwithstanding other parts of Africa and the world at large. Njogu et al. [42] investigated the antidiabetic activity of *K. africana* aqueous and ethyl acetate extracts using male Swiss albino mice. Hyperglycemia was induced experimentally by a single-dose intraperitoneal administration of 186.9 mg/kg body weight of a freshly prepared 10% alloxan monohydrate [79]. After forty-eight hours, mice with blood glucose levels above 200 mg/dL were considered diabetic and used in this study. The mice were randomly divided into seven groups of five animals each. Group 1 consisted of normal mice either intraperitoneally or orally administered with 0.1-mL physiological saline, group 2 consisted of alloxan-induced diabetic mice either intraperitoneally or orally administered with 0.1-mL physiological saline, group 3a consisted of alloxan-induced diabetic mice intraperitoneally administered with 0.025 insulin units (0.25 insulin units in 1 mL) (1 IU/kg body weight) in 0.1-mL physiological saline, group 3b consisted of alloxan-induced diabetic mice orally administered with 0.075-mg glibenclamide (0.75 mg in 1 mL) (3 mg/kg body weight) in 0.1-mL physiological saline, group 4 consisted of alloxan-induced diabetic mice either intraperitoneally or orally administered with 1.25-mg extract (12.5 mg in 1-mL physiological saline) (50 mg/kg body weight) in 0.1-mL physiological saline, group 5 consisted of alloxan-induced diabetic mice either intraperitoneally or orally administered with 2.5-mg extract (25 mg-extract in 1-mL physiological saline) (100 mg/kg body weight) in 0.1-mL physiological saline, group 6 consisted of alloxan-induced diabetic mice administered with 5-mg extract (50-mg extract in 1-mL physiological saline) (200 mg/kg body weight) in 0.1-mL physiological saline and group 7 consisted of alloxan-induced diabetic mice either intraperitoneally or orally administered with 7.5-mg extract (75-mg extract in 1-mL physiological saline) (300 mg/kg body weight) in 1-mL physiological saline. A volume of 0.1 mL of either insulin or glibenclamide or the plant extract solution was administered either intraperitoneally or orally to each experimental mouse. The same experimental design was adapted for organic fractions, too. The selected dosages were calculated as 2 log doses between 50 and 300 mg/kg body weight based on acceptable therapeutic doses for bioscreening. The alloxan-induced diabetic rats had a three to four-fold increase in blood glucose relative to the normal control rats. The aqueous and ethyl acetate leaf extracts of *K. africana* showed a blood glucose-lowering effect when administered intraperitoneally and orally, an indication that they contained hypoglycemic constituents. 

#### 3.4.4. Antiprotozoal Activity 

According to the latest World Malaria Report, released in November 2018, there were 219 million cases of malaria in 2017, up from 217 million cases in 2016. The estimated number of malaria deaths stood at 435,000 in 2017, a similar number to the previous year [80]. The WHO African region carries a disproportionately high share of the global malaria burden. In 2017, the region was home to 92% of malaria cases and 93% of malaria deaths [80]. The wood extract of *K. africana* has been reported to possess antimalarial activity against drug-resistant strains of *Plasmodium falciparum* superior to that of chloroquine and quinine [81]. Atawodi and Olowoniyi [60] reported the efficacy of hexane, dichloromethane, ethyl acetate and ethanol extracts of *K. africana* root bark against *P. falciparum* [82] and *Trypanosoma brucei* and *Trypanosoma brucei rhodesiense* [58], the causative organisms for malaria and sleeping sickness, respectively. Bharti et al. [61] also reported that the growth of *Entamoeba histolytica* was inhibited by the stem bark butanol extract of *K. africana*. Four compounds exhibiting significant antiplasmodial activity were isolated from the ethyl acetate extract of *K. africana*. Three of the four isolated compounds showed good activity against all the different parasite strains, the chloroquine resistant W-2 and two field isolates of *P. falciparum*, with IC50 < 5 µM. Specioside exhibited the highest activity on W-2 (IC50 = 1.5 µM), followed by 2β, 3β, 19α-trihydroxyurs-12-en-28-oic acid (IC50 = 1.60 µM) and atranorin (IC50 = 4.41 µM), while p-hydroxycinnamic acid was the least active (IC50 = 53.84 µM) [48].

Lapachol in the methanol extract of the root and a quinone obtained from the wood showed antimalarial activity. Three iridoids, specioside, verminoside and minecoside isolated from the butanol extract of the stem bark possess antiamoebic activity [83]. The antitrypanosome activity of the stem bark and root bark extracts are attributed to 2-(1-hydroxyethyl)-naphtho-[2,3-b]-furan-4,9-quinone and three naphthoquinoids: isopinnatal, kigelinol and isokigelinol [58].

Akeng’a Ayuko et al. [84] investigated the antiplasmodial activity of *K. africana* chloroform, methanol and ethyl acetate extracts against two *P. falciparum* strains, viz., chloroquine (CQ)-sensitive *P. falciparum* from Sierra Leone (D-6) and CQ-resistant *P. falciparum* from Vietnam (W-2). CQ was used as a positive control. An in vitro semiautomated microdilution assay technique was used to measure the ability of *K. africana* to inhibit the incorporation of [G-3H] hypoxanthine into the malaria parasite [85,86]. Aliquots of the culture medium (25 µL) were added to all the wells of a 96-well flat-bottomed microtiter plate. The test solutions (25 µL) were added in duplicate to the first wells, and a motorized hand diluter was used to make two-fold serial dilutions of each sample over a 64-fold concentration range. The susceptibility tests were carried out with an initial 200 µL of parasite culture (0.4% parasitemia and 1.5% hematocrit) in each well. The stock solutions of *Kigelia* extracts were diluted in the plate to give a 100-µg/mL concentration (as the highest concentration) and then diluted two-fold, until reaching a concentration of 1.56 µg/mL. A suspension (200 µL, 1.5% *v*/*v*) of parasitized erythrocytes (0.4% parasitemia) in the culture medium was added to all the test wells. Nonparasitized erythrocytes were used in control experiments. The plates were incubated at 37 °C in an atmosphere of 3% CO_2_, 5% O_2_ and 92% N_2_. After 48 h, each well was pulsed with 25 µL of culture medium containing 0.5 µCi of [G-3H]-hypoxanthine, and the plates were incubated for a further 18 h. The contents of each plate were harvested onto glass fiber filters, washed thoroughly with distilled water, dried and the radioactivity measured using a Beta-plate TM liquid scintillation counter (Wallac, Zurich, Switzerland). The activities of the crude extracts were grouped according to Deharo et al. [87]. Thus, any extracts that showed an IC_50_ for antiplasmodial activity of less than 5 µg/mL were considered active, while those that had IC_50_ values from 5–10 µg/mL were considered moderately active, and those extracts with IC_50_ values over 10 µg/mL were considered inactive. The activities obtained indicated that there were differences in the strains’ sensitivities to the extracts. The chloroform, methanol and ethyl acetate stem bark extracts of *K. africana* were found to be inactive in vitro with CQ-sensitive IC_50_ values of 29.01 ± 0.78, 4.50 ± 0.04 and 25.77 ± 0.30, respectively, and had CQ-resistant IC_50_ values of 16.79 ± 0.94, 22.63 ± 0.95 and 27.84 ± 1.29, respectively. Together with previous researchers reporting *Kigelia* as a good antiplasmodial agent, coupled with its common traditional use for malaria, this study is quite fascinating. Nonetheless, several factors affect the potency of a potentially active plant extract when tested. These factors range from poor environmental conditions, poor harvesting and handling practices, wrong drying methods used, poor experimental design, poor extractant solvent concentrations and poor stock preparation, to mention but a few. Additionally, the degradation of active compounds during storage or the presence of prodrugs that undergo enzymatic transformations in vivo to give active antiplasmodial compounds are also factors to consider [88]. 

#### 3.4.5. Antiurolithiatic Activity

The third-most common disorder of the urinary tract is urolithiasis after urinary tract infections and benign prostatic hyperplasia [89]. The worldwide incidence of urolithiasis is quite high, and despite tremendous advances in the field of medicine, there is no truly satisfactory drug for the treatment of renal calculi [90]. Most patients still must undergo surgery to get rid of this painful disease. Hyperoxaluria is the main initiating factor for urolithiasis, and most calculi in the urinary system arise from a common component in urine, e.g., calcium-oxalate (CaOx), representing up to 80% of analyzed stones [91]. In West Africa, *K. africana* leaves are used in the treatment of kidney ailments [16]. This has been proven in various studies. According to the authors of [92], the aqueous *K. africana* fruit extract can alkalinize urine, making it less acidic. It has shown significant antiurolithiatic activity in the dissolution of generated calcium oxalate crystals. Gupta et al. [93] reported that the antiurolithiatic activity of *K. africana* fruit extract may possibly be mediated through the inhibition of calcium oxalate crystallization, making the extract curative, as well as having prophylactic uses in urolithiasis. In another study, the effect of the ethanolic extract of *K. africana* fruit on calcium oxalate urolithiasis was studied in male Wistar albino rats. The ethylene glycol feeding resulted in hyperoxaluria, as well as increased renal excretions of calcium, magnesium and phosphate. Supplementation with the ethanolic extract of *K. africana* fruit significantly reduced the elevated urinary oxalate, uric acid and phosphate. The ethanolic extract of *K. africana* fruit also significantly lowered the increased deposition of stone-forming constituents in the kidneys of calculogenic rats. This is an indication that the ethanolic extract of *K. africana* fruit possesses urolithiatic activity [93].

#### 3.4.6. Anticonvulsant Activity

*Kigelia africana* aqueous and methanolic bark extracts were investigated for anticonvulsant activity in Wistar rats using pentylenetetrazol (PTZ) and maximal electroshock (MES)-induced convulsions method. Both the extracts demonstrated potent anticonvulsant activity, which is due to the presence of linoleic and cinnamic acid. Doses of 250 mg/kg and 500 mg/kg of methanolic and aqueous extracts were administered to the rats intraperitoneally. The extracts gave significant protection against the PTZ and MES-induced convulsions [94].

#### 3.4.7. Antidiarrheal Activity

Indigenous knowledge of the use of bark, leaves and fruits of *K. africana* as a remedy for diarrhea among African communities has been documented by several researchers (Table 1). Owolabi and Omogbai [95] investigated the antidiarrheal properties of *K. africana* ethanolic bark extracts using Swiss albino mice. Five treatments were given. Group 1 (negative control) was treated with normal saline (10 mL/kg), and group 5 (positive control) received atropine sulphate (0.1 mg/kg). Groups 2, 3 and 4 received the ethanolic bark extract (100, 200 and 500 mg/kg, respectively). Castor oil was orally administered to induce diarrhea in the mice (0.3 mL). In order to enhance small intestinal motility, the mice were fasted overnight. Results of this study showed that oral doses of 100, 200 and 500 mg/kg caused a marked inhibition of the diarrhea response following the castor oil administration (*p* < 0.05). *Kigelia* africana ethanolic bark extracts significantly (*p* < 0.0001) inhibited the small intestinal motility in mice, with the 500 mg/kg dose giving the highest effect in both castor oil-induced diarrhea and small intestinal motility. When compared with the positive control, atropine, the antidiarrheal effect of *K. africana* ethanolic bark extracts at 500 mg/kg was 82% and 62.7%, respectively, on castor oil- induced diarrhea and small intestinal motility. This experiment was well-designed. The negative and positive controls are suitable for this study. Whereas the LC_50_ was given, the authors would have prepared the lowest dose where there were no visible side effects. This is to help establish a no-effect dose, which is relevant in the case of further chronic studies and to establish an effective range in the case of efficacy studies. This is majorly because, if the first tested dose is effective and, following this, the LD_50_ is established, how can we establish the starting effective dose or the range if we do not start from the lowest? According to the Organisation for Economic Co-operation and Development 408 (2018) guidelines, a descending sequence of dose levels should be selected with a view to demonstrating any dosage-related response and a no-observed adverse-effect level (NOAEL). Furthermore, the authors were not specific on the sex of animals used in the study. Whereas, in the beginning, they stated that the animals were of either sex, they did not elaborate on the number of animals used per sex which is a deficiency. OECD guidelines emphasize the use of both sexes (male and female) in experiments, unless in cases where the pharmacokinetics of the drug are specific to the physiology of the animals. The authors should have evaluated the effects of this treatment at the sex level and investigated whether any variations exist due to the differences in body physiology. 

#### 3.4.8. Treatment of Sexually Transmitted Diseases

Traditional healers in the Igbo tribe in South-Eastern Nigeria use an aqueous or dilute alcohol extract of *K. africana* rootbark as a treatment for sexually transmitted diseases. Root extracts equivalent to those used in traditional preparations were found to contain the iridoids specioside and minecoside as major constituents. The root extracts, as well as two of the isolated iridoids, were tested, and their 1/10 and 1/100 dilutions were tested against four bacteria species, viz., *Staphylococcus aureus*, *Bacillus subtilis*, *Escherichia coli* and *Pseudomonas aeruginosa* and the yeast *Candida albicans*, both in the absence and presence of the enzyme emulsin. Emulsin enzyme converts catalpol-type iridoids to their more antimicrobially active non-sugar-containing aglycones. The growth of the organisms in culture broth was assessed by measuring the turbidity of the solution [96]. The results showed that the aqueous extract had strong activity, even in the absence of emulsin, against all the bacteria tested but, especially, against the yeast *C. albicans*. *Candida* infections are common opportunistic infections of the genito-urinary tract, and the traditional use of this plant extract might alleviate this in sexually transmitted diseases. Akunyili et al. [96] stated that the root extracts tested were equivalent to those used in traditional preparations, but they did not mention what concentrations were used. It was not mentioned how root extracts were prepared traditionally—for example, the ratio of water to the root powder and related information. This raises the concern that the experiment cannot be reproduced, and the results may not be reliable.

*Kigelia africana* root extracts were reported as an effective treatment for sexually transmitted diseases, and this was concluded from a broth dilution susceptibility study against *Candida albicans* [97]. This pharmacological study, however, does not provide evidence of the successful treatment of *C. albicans* in humans. Broth dilution is a susceptibility test that can be very inaccurate, especially as individual mistakes in the preparation of stock concentrations can occur, and automation is impossible. The controls in this study are not mentioned. Most researchers employ in vitro tests at the beginning of their studies, and it is a good start to give an indication of the activity of any biological material. Nonetheless, in vitro studies must always be followed by a series of in vivo studies in biological systems to determine their clinical effectiveness. In vivo studies have higher importance, since they indicate the pharmacokinetic and pharmacodynamic characteristics of the materials under investigation. Additionally, detailed knowledge of the chemical formulations, administrative pathways and dosage used is critical when investigating and reporting an in vivo study. 

#### 3.4.9. Diuretic Activity

Sharma et al. [98] investigated the diuretic activity of *K. africana* aqueous bark extract by determining the urine volume, electrolyte concentration and diuretic potency in male albino rats. Different concentrations of the extract, 250 and 500 mg/kg were orally administered to hydrated rats, and their urine output was immediately measured after five hours of treatment. Furosemide (10 mg/kg) was used as the reference drug, while normal saline (0.9%) solution was used as the control. The result showed that the bark extract exhibited a dose-dependent diuretic property. The onset of diuretic action was within one hour and lasted up to five hours, with 500 mg/kg displaying more activity than 250 mg/kg. The extract also caused a marked increase in Na^+^, K^+^ and Cl^−^ labels [99].

#### 3.4.10. Antioxidant Activity

According to Ponnan et al. [100], antioxidant compounds are abundantly available in plants and play an important role in scavenging free radicals, thus providing protection to humans against oxidative DNA damage. An excess of reactive oxygen species (ROS) can result in noncontrolled oxidation (oxidative stress) and damage of cellular structures, such as DNA, protein and membrane lipids. It is believed that the presence of ROS is essential in cells, as they can act as key signaling molecules for the activation of the stress responses and defense pathways [101]. The antioxidant activity of *K. africana* has been investigated by various researchers, and *K. africana* plant parts have proven to have good antioxidant activity. Hussain and colleagues investigated the antioxidant activity of *K. africana* bark, fruit and leaf using a DPPH (1,1-diphenyl-2-picrylhydrazyl) free radical assay [102]. Quercetin was used as a standard antioxidant, which showed 94% inhibition. The bark of *K. africana* possesses good antioxidant activity (67.33%); the fruit extract showed moderate antioxidant activity (62.66%), and the leaves showed the poorest antioxidant activity (59.66%), respectively [16]. In another study, the free radical scavenging ability of *K. africana* against DPPH was evaluated [103]. Ascorbic acid was used as a control. The methanolic stem bark extract scavenged DPPH in a dose-dependent manner with IC_50_ = 175 µg/mL. *Kigelia africana* significantly (*p* < 0.05) scavenged the DPPH stable radical at IC_50_ = 175 µg/mL by 16.75%, 27.57%, 38.28% and 53.2%, respectively, when compared to ascorbic acid [33]. 

According to Olubunmi et al. [18], the free radical scavenging activities of *K. africana* root extract through the spectrophotometric assay on the reduction of DPPH compared favorably with α-tocopherol (standard antioxidant) at high concentrations. Scavenging activity was observed for the root extract at all concentrations (100, 250, 500 and 1000 μg/mL) assayed, with 250 μg/mL having the lowest activity, while the highest antioxidant capacity was observed at 1000 μg/mL. Dhungana et al. [11] reported the antioxidant activity of the methanolic leaf and fruit extracts of *K. pinnata*. *Kigelia pinnata* extracts showed a significant reduction in free radical-related complications, lipid peroxidation, blood cholesterol and low-density lipoproteins [104]. In this study, the ethyl acetate fraction of the plant root had high antioxidant activity against DPPH, which may be due to the presence of a high phenolic content [36]. An ex-vivo assessment of the antioxidant property of *K. africana* extracts in rat liver homogenate was carried out by Olaleye and Rocha [105]. Administration of different pro-oxidants: 10-µM iron (II) sulphate, (FeSO4), 5-µM sodium nitroprusside (SNP) and 2-mM 3-nitropropionic acid led to an increased formation of thiobarbituric acid-reactive substances (TBARS), which indicates lipid peroxidation in the liver. Administration of *K. africana* methanolic fruit extracts significantly (*p* < 0.05) reduced the production of TBARS in a concentration-dependent manner in all the pro-oxidant-induced oxidative stress, suggesting that the use of the plant in the treatment of various diseases, especially liver diseases, could be due to its ability to act as an antioxidant [105].

Several researchers have conducted in vitro antioxidant assays on *K. africana* and many other medicinal plants. Whereas the in vitro DPPH assay is commonly used to evaluate the radical scavenging activity of plant extracts [11,16,18,103], it is not a measure of pharmacological activity. Moreover, the DPPH radical is synthetic and not biologically relevant, implying it does not mimic any known free radical produced in biological systems. Therefore, to conclude on the antioxidant status in a given sample through in vitro assays, ABTS (2,2’-azino-bis (3-ethylbenzothiazoline-6-sulphonic acid), FRAP (Ferric-Reducing Ability of Plasma) and ORAC (Oxygen Absorbance Capacity) assays should be used in combination with DPPH. Of all the methods, ABTS is the most sensitive [106]. Furthermore, in the reviewed studies, ascorbic acid was the only control used. However, in order to increase the validity and reliability of in vitro antioxidant results, at least two positive controls—for example, Trolox, Vitamin E, Vitamin C and BHT (butylated hydroxytoluene)—must be used in antioxidant assays. In any scientific experiment, using controls increases the reliability of the results, since often a comparison is made between control measurements and the other measurements. In the reviewed studies, whereas the authors mention the controls used in the antioxidant assays, they do not mention their concentrations. This, in turn, reduces the reliability of the study results. Generally, antioxidant activity is a chemical activity and can be found in any plant, and without proper in vivo studies, including bioavailability studies, antioxidant assays cannot relate to any medicinal use. Moreover, in recent literature, doubt has been expressed about the pharmacological significance of antioxidants. In vivo studies are always required, including proper positive and negative controls [101,107].

#### 3.4.11. Anticancer Activity 

According to Khan and Mlungwana [108], the anticancer potential of *K. africana* has been indicated by cytotoxicity of the root and bark materials in the brine shrimp bioassay against *Artemia salina*. Houghton and colleagues reported significant inhibitory activity of stem bark extracts against four melanoma cell lines and a renal carcinoma cell line and slight activity by fruit extracts [109]. The root bark had activity against KB cells [82]. Inhibitory effects of *K. africana* fruit extracts on induced tumors and inflammation in mice have been reported [110]. Momekov et al. [111] investigated the anticancer activity of *K. africana* methanolic stem bark extract. The powdered (1 mm) stem bark was refluxed with methanol (1: 20) for 1 h at 80 °C. After cooling at room temperature, the extract was filtered, and the residue was subject to the same extraction process twice. Thereafter, the filtrates were gathered, and the solvent was evaporated in vacuo to dryness. The methanolic extract of *K. africana* stem bark had significant cytotoxicity against human tumor cell lines, with IC_50_ values of 15.1 ± 3.4, 126.0 ± 9.1, 90.7 ± 4.7, 186.0 ± 9.2, 101.0 ± 7.4, 124.1 ± 8.9, 11.8 ± 3.8 and 10.2± 2.7 µg/mL against T-cell leukemia (a KE-37 derivative), acute lymphoid leukemia, acute myeloid leukemia, chronic myeloid leukemia, non-Hodgkin’s lymphoma, Hodgkin’s lymphoma, breast cancer and murine lung cancer cell line, respectively. These results compared with the vincristine-positive control (IC_50_ = 0.22 ± 0.1, 3.3 ± 0.9, 1.5 ± 0.7, 4.1 ± 1.4, 1.2 ± 0.6, 7.4 ± 1.0, 3.7 ± 0.6 and 2.2 ± 0.4 µg/mL) against T-cell leukemia (a KE-37 derivative), acute lymphoid leukemia, acute myeloid leukemia, chronic myeloid leukemia, non-Hodgkin’s lymphoma, Hodgkin’s lymphoma, breast cancer and murine lung cancer cell line, respectively [111]. Momekov et al. [111] carried out an extremely useful study, although cytotoxicity of the extracts on normal cells was not reported. This leaves the reader wondering whether the safety of the extracts on normal cells was first established before testing the extracts on cancer cell lines. Testing plant extracts on normal cells is of paramount importance before tests on cancer cell lines are conducted. This is majorly because, whether an extract is toxic on cancer lines but not safe on normal cell lines, it is not a good product lead. Therefore, nonselectivity is an issue that was not addressed by the authors.

In another study, the antitumor activity of *K. africana* methanolic leaf extracts of 100 and 200 mg/kg were evaluated against the Ehrlich ascites carcinoma (EAC) tumor induced into mammary glands of mice. Acute and short-term toxicity studies were performed at the beginning to ascertain the safety of methanolic extracts. After 12 days of tumor inoculation, the extract was administered daily for 30 days. The effect of the methanolic extracts of *K. africana* on the growth of the tumor, life span of EAC-bearing hosts and simultaneous alterations in the hematological and histopathological profile were estimated. The methanolic extracts of *K. africana* resulted in a decrease in the tumor size and improved average body weight and mean survival time, thereby increasing the life span of EAC tumor-bearing mice [63].

Higgins and colleagues investigated the cytotoxic activity for *K. africana* fruit extracts against melanoma and two breast cancer cell lines [112]. They used a bioactivity-driven separation approach to identify demethylkigelin, kigelin, ferulic acid and 2-(1-hydroxyethyl)-naphtho[2,3-b] furan-4,9-dione as the compounds thought to be responsible for the cytotoxicity. Of these, 2-(1-hydroxyethyl)-naphtho[2,3-b] furan-4,9-dione was a particularly potent cytotoxic agent. 

Potent antiproliferative activity against the Caco-2 and HeLa carcinoma cell lines was noted for *K. africana* methanolic fruit extracts [45]. Of further interest, the chloroform and hexane *K. africana* fruit extracts also demonstrated a stimulatory effect on cell proliferation. The root bark extracts of *Kigelia* have been recommended to treat uterine cancer [113]. Chivandi and colleagues investigated the effect of *K. africana* seed oil on cell proliferation in the culture using human colon adenocarcinoma (Caco-2) and human embryonic kidney (HEK-293) cells [114]. They maintained and treated the cells with various concentrations (0, 20, 40, 80, 100 and 120 mg/L) of *K. africana* seed oil. The trypan blue dye exclusion method was used to determine cell growth 48 hours after oil treatment. The study results showed that the *K. africana* seed oil suppressed both Caco-2 and HEK-293 cell growth in a dose-dependent manner. The oil did not increase cell death, as the number of dead cells remained unchanged under control and oil-treated conditions. The oil significantly suppressed Caco-2 cell growth at all concentrations compared to HEK-293 cell growth. Whereas the Caco-2 cell line is a continuous line of heterogeneous human epithelial colorectal adenocarcinoma cells, HEK293 cells are immortalized by Ad 5 E1A and E1B. HEK293T cells also express SV40 large T antigen. Thus, the cells lack normal pRb and p53 functions. The suppression of Caco-2 and HEK-293 cell proliferation by *K. africana* seed oil suggests a potential antiproliferative effect of the oil on the two cell lines [114]. The methanolic extract of *K. africana* root contains the constituent lapachol [115], which is reported to be effective in the treatment of solar keratosis, skin cancer and Kaposi sarcoma, an HIV-related skin ailment [47]. 

*Kigelia africana* has also found application in traditional cancer therapy, and some studies support its cytotoxic effects on melanoma, Ehrlich ascites carcinoma and breast cancer cells. Unfortunately, there a few reports regarding the IC_50_ of *K. africana* on normal cells, which leaves one contemplating whether this species is selectively toxic to cancer cells when taken as a remedy. Furthermore, there is a need to do more in vivo studies regarding the biological activity of *K. africana,* as this would provide a clear picture of the pharmacokinetics and pharmacodynamics of the targeted compounds in biological systems. 

Several researchers have concluded that, if a plant has a cytotoxic effect, it has anticancer properties, which may not be correct. It is only after positive clinical trials for cancer treatment that any substance or compound can be confirmed anticancer. Another dilemma is most often that the terms cytotoxic, anticancer and chemopreventive are used interchangeably, despite each having a completely different meaning. Whereas an anticancer agent is any substance that is effective in the treatment of malignant, or cancerous, diseases, a chemopreventive agent is any natural, synthetic or biologic chemical agent that reverses, suppresses or prevents carcinogenic progression to invasive cancer [116]. Cytotoxic agents are substances that contain chemicals that are toxic to cells, preventing their replication or growth. Additionally, most often, researchers use the term cytotoxicity to mean anticancer, which is misleading. A cytotoxic agent is not limited to use in cancer treatments but can also be used to treat several other disorders, such as rheumatoid arthritis and multiple sclerosis. In a nutshell, the activity of herbal preparations is not a function of a single compound but, rather, a mixture of compounds that act in synergy to cause an effect. Until the active compound responsible in a given herbal preparation is identified, isolated and tested, plant extracts simply remain effective subject to purification and standardization. 

#### 3.4.12. Toxicological Evidence

Sharma and colleagues went further to investigate the toxicity of the aqueous bark extract using experimental rats and found that it was safe up to 5 g/kg [98]. The acute toxicity of the *Kigelia* methanolic fruit extract was investigated using male Sprague–Dawley rats. In this study, the extract was well-tolerated by the animals, as there were no observable signs of acute toxicity like restiveness, seizure or dizziness after the administration of 400 mg/kg. However, at 6400 mg/kg, the animals showed signs of toxicity like jerks and writhes with 60% death. At 12,800 mg/kg, there was 80% death of the animals. The LD_50_ was estimated from a log dose curve to be 3981.07 mg/kg [38,51]. In another study, 100 mg/kg aqueous extract was administered to rats induced with acetaminophen liver toxicity. The extract countered the effect of acetaminophen on the activities of aspartate transaminase (AST), alanine transaminase (ALT), superoxide dismutase (SOD), catalase (CAT), gluthathione peroxidase (GPx) and δ- amino levulinate dehydrogenase (δ-ALAD) [105]. 

Nyarko and colleagues conducted a comparative study of subchronic and chronic toxicity of *K. africana* using various combinations [117]. An aqueous antidiabetic polyherbal extract ADD-199 containing *K. africana* and three other plants at a daily dose of 100 or 500 mg/kg body weight were administered to male Wistar albino rats over 30 days. After the 30-day study, there was no effect on the hematological, urinary and plasma biochemical parameters. The extract also had no effect on some modulators of hepatic cytochrome P450 (CYP) isozymes normally measured as indices of organ-specific toxicity or the potential for drug interactions. Specifically, ADD-199 containing *K. africana* did not affect the plasma AST, ALT, alkaline phosphatase (ALP) and albumin or creatinine kinase (CK) levels. It also did not affect plasma creatinine and urea levels. Furthermore, ADD-199 neither affected the packed cell volume (PCV) nor the levels of red blood cells (RBC), reticulocytes, platelets, lymphocytes and granulocyte. It, however, caused significant dose-dependent reductions in white blood cell counts at day 15, with varying degrees of recovery by day 30. ADD-199 also reduced the rate of body weight increases in week 3. However, no changes were observed in organ weight at termination. The ADD-199 did not significantly affect zoxazolamine-induced paralysis and pentobarbital-induced sleeping times, as well as certain CYP isozyme activities in rats, suggesting that ADD-199 had no overt organ-specific toxicity and did not demonstrate a potential for drug interactions via CYP-mediated metabolism in rats following subchronic administration. 

The protective effect of the methanol extract of the *K. africana* fruit extract against cisplatin-induced renal toxicity in male rats was investigated by Azu et al. [38]. The rats treated with cisplatin for 28 days suffered a loss in body weight, elevation in blood urea nitrogen and serum creatinine levels, as well as tubular necrosis. Pretreatment with the *K. africana* fruit methanol extract at 100 mg/kg as a prophylaxis significantly prevented these changes. Though the posttreatment of animals with the extract after a cisplatin treatment did not completely restore the serum catalase activity, it caused some alleviating effects, suggesting that the *K. africana* fruit extract may protect against cisplatin-induced renal toxicity and, hence, might serve as a novel agent to limit renal injury.

Studies on the pharmacological activity of *K. africana* in Section 3.4 are summarized in Table 3 below.

As per the reviewed studies, the authors investigated the biological activity of *K. africana* as a single species, whereas most traditional medicine preparations usually involve the use of *K. africana* in combination with other plant species (Table 1). This is due to the effectiveness of polyherbal medicine preparations compared to single plant preparations. Therefore, the polyherbal concept should be adopted by researchers when carrying out laboratory experiments to get an understanding of the effects of molecules from different plants (synergy) and their mechanisms of action, as compared to the use of a single species. On the other hand, if a single species is investigated, as it is often done, researchers should understand that the efficacy of any herbal remedy is not a function of a single compound. It involves the synergy and antagonism of several compounds to induce activity. This is one of the many reasons isolated plant compounds should be investigated for synergism and antagonism to understand how different compounds interact to cause effects.

## 4. Conclusions

Despite efforts by several researchers to document the traditional uses of *K. africana* (Table 1), a lot of information has been lost, owing to the death of custodians commonly known as living libraries. In addition, many traditional uses have not been scientifically validated; thus, they are simply claims. Thus, the gap to completely profile the ethnobotanical knowledge and phytochemistry and pharmacological activity of *K. africana* is still wide, and more research needs to be conducted to discover the unknowns and confirm the knowns. This will increase the significance of this species at the international level, as compared to the community level. *Kigelia africana* contains many phytochemicals that have already been identified (Table 2), isolated and their pharmacological activity validated. Nonetheless, the mechanism of action for pure compounds has not been studied for the majority. As per the reviewed studies, most pharmacological studies that have been carried out on *K. africana* plant parts have been based on traditional uses [12]. Fruits have received more attention from researchers regarding their bioactivity compared to other plant parts. This has left little scientific basis for the bioactivity of the leaves, flowers, stems and roots [18]. Therefore, other plant parts need to be given similar attention, since they may have unique and highly potent phytochemicals.

Despite the norms surrounding herbal medicine preparations as being safe with fewer side effects [118], this is completely wrong, except when proven in the laboratory. Many herbal medicine preparations have caused life-threatening side effects and death in the worse scenarios [119]. Although many such cases have not been documented, some cases of poisoning have been reported in the literature [120,121,122]. Therefore, quality, efficacy and safety are key elements to consider before using any herbal product or making it commercially available [123]. Unfortunately, several products have been formulated from *K. africana* and are available on the market, yet they do not meet international quality standards. This implies that most of these product formulations have no standard dose and are of uncertain quality, efficacy and safety. Thus, if the availability and acceptance of *K. africana* products on international markets is to be increased, programs to promote training on efficacy, safety, international quality standards, sustainable use and conservation of the natural resource base need to be established. 

For a brighter future of medicinal plant research, researchers should embrace a high-throughput analysis before coming to conclusions concerning the biological activity of medicinal plants. This is because a high-throughput analysis combines genomics, proteomics and chemical and ultrastructural data. Additionally, all in vitro studies need to be followed by clinical trials to demonstrate the safety and efficacy of traditional treatments in biological systems, and all experiments must comply with international scientific standards and guidelines. There is also a need to add more information to the basic pharmacological assays and aim for clinical trials by focusing on molecular drug and disease targets [124]. 

Traditional medicine (TM) is strongly embedded in indigenous systems that are rooted within local communities and are very strong. Despite the strong roots of TM, there is a huge stumbling block to its development, and this is ignoring the capacity and roles of TM practitioners in national and international policies. Additionally, physicians and other health professionals are also not exposed to TM in their training, leading to a wide gap between conventional and TM practice and a disconnect between professional health groups and patients who choose to continue TM use. Another stumbling block to TM development is the failure of researchers to guard the intellectual property of local communities where they collect indigenous knowledge (IK) for their research. These two stumbling blocks are of regional, national and international importance. Interestingly, intellectual property rights frameworks such as the access to genetic resources and benefit sharing exist, but they are not fully operational, presenting a bottleneck for equitable benefit sharing, which is a prerequisite in fostering partnerships [125]. Therefore, researchers should take on the mantle and jealously guard the intellectual property (IP) of indigenous communities through inclusive patenting procedures. Similarly, the principle of prior informed consent as enshrined in the Convention for Biological Diversity needs to be enforced while collecting ethnomedical information.

## 5. Limitations

This review provides an overview of the published developments on *Kigelia af ricana*, with the aim to discuss its current context in Africa. As such, this paper does not present a systematic review, nor does it present a meta-analysis of all primary and secondary literature published on the topic areas of interest. When synthesizing findings from technical reports and standard testing guidelines published by leading international organizations such as WHO, EUCAST and OECD, respectively, the review relied on the original authors’ quality assessments of studies and instructions included within their reports. 

## Figures and Tables

**Table 1:** Traditional uses of *Kigelia africana* (Lam.) Benth. in Africa.

Region/Country	Plant Part/Preparations	Traditional Use	Reference
South Africa	Fruits	Solar keratosis, malignant melanoma, dysentery, worm infestations, pneumonia, toothache, malaria, diabetes, venereal diseases, convulsions, antidote for snakebite, postparturition hemorrhage, solar keratoses and skin cancer	[3,6,9,11,20,21]
	Roasted seeds	Pneumonia, fungal infections, eczema, malaria, diabetes and waist pain	[6,22]
	Stem and root bark	Ulcers, pneumonia and toothache	[23]
South Africa and Zimbabwe	Fruits	Crude fruit creams for freckles	[24]
South Africa and Ethiopia	Hot root macerate	Gynecological complaints, constipation and tapeworm infections	[6]
	Root bark	Uterine cancer, venereal diseases, hemorrhoids and rheumatism	[6,25]
	Stem bark	Rheumatism, dysentery, venereal diseases, gynecological conditions, hemorrhages, epilepsy, wounds, sores, abscesses, diarrhea and edema	[6]
South Africa and Cameroon	Stem bark decoction mixed in porridge	Infertility	[23,26]
South Africa and Namibia	Stem and leaves decoction	Eczema and herpes	[27]
	Fruits and stem bark decoction	Worm infections in children	[22]
Zambezi Valley	Fruits	Crude fruit cosmetic preparation used by Tsonga women, dressing for ulcers, purgative and galactagogue	[22,23]
Zambia	Bark	Syphilis and gonorrhea	[23]
Botswana	Fruits boiled with milk	Sexually transmitted diseases	[22]
West Africa (general)	Leaves	Gastrointestinal ailments	[22,28]
	Bark water macerate	Antidote for snakebite, sores, skin fungal infections, dysentery and syphilis	[29,30]
	Ground bark and fruit infusion	Stomach problems in children	[22]
	Root and bark	Pneumonia, tapeworms, ulcers and gynecological complaints	[15,22,31]
	Fruits	Wounds, abscesses, antimalaria, febrile jaundice and menorrhagia	[27,32]
	Aqueous bark extract	Backache, stomach pains and dysentery	[32]
	Leaves and twigs	Constipation, gynecological disorders, hemorrhoids, lumbago, dysentery, wounds kidney disorders, snakebite and rheumatism	[22,27]
	Leaves	Stomach and kidney ailments, antidote for snakebites and wounds	[16,27]
	Stem and twigs	Wounds, antidote for snakebites, rheumatism, stomach and kidney ailments	
	Fruits, roots and leaves	Sexual complaints, viz., poor libido, sexual asthenia and impotence	
	Fruits	Dermatitis—fruit ointment, constipation, gynecological disorders, hemorrhoids, psoriasis, eczema, diarrhea, malaria, rheumatism, retained placenta, dressing for wounds, purgative, galactagogue and dizziness	[16,22,25,33,34]
	Bark	Antimicrobial, cytotoxicity and anti-implantation activities	[3,22,35,36]
Cameroon	Stem bark decoction	Abortifacient, filariasis and cataract	[22]
Ghana	Bark preparation	Dysentery and rheumatism	[37]
Togo	Fruits	Cancer	[9]
Ivory Coast	Fruit infusion	Rheumatism and back pains	[22]
Benin, Ivory Coast, and South Africa	Leaf decoction	Jaundice	[6]
Nigeria	Bark	Anti-inflammatory, dysentery and anticancer	[5,12,37]
	Fruits	Psoriasis, eczema, leprosy, rheumatism, snakebites, syphilis and chronic abdominal pain	[38,39]
	Root decoction	Ante and postnatal disorders, fibroid and conception	[6]
	Fruits and flowers mixed with alcohol or water	Fertility treatment among women and men of childbearing age	[36]
	Leaves	Diarrhea, abortifacient, aphrodisiac, tonic and impotence	[40]
Central Africa	Unripe fruit	Dressing for wounds, hemorrhoids and rheumatism	[6,39]
Kenya	Roasted seeds mixed with beer	Enlargement of sexual organs	[23,38]
Kenya, Embu community	Fruits	Cold, flu, inflammation and dysentery	[41]
Tanzania	Stem bark infusion	Hyperpyrexia and gonorrhea	[6]
	Fruits boiled	Anemia, especially in pregnant women	
East and West Africa	Bark	Convulsions	[9]
Tanzania and Nigeria	Hot decoction of stem bark	Galactagogue	[26]
Africa (general)	Bark decoction	Laxative	[27]
	Ash leaves mixed with honey	High blood pressure	[22]
	Fruits	Mature fruit is used for treating wounds, abscesses, dressing wounds, skin cancer, reducing breast metastasis, ulcers, syphilis, rheumatism, fungal infections, boils, psoriasis, leprosy, venereal diseases and acne	[3,14,22,23,27,35,42]
	Leaves	Malaria, rheumatism, wounds, ulcers, retained placenta, venereal diseases and diarrhea	[43]
	Fruit and bark (lesser extent) extracts	Dysentery, hemorrhoids, constipation, wounds, ulcers, boils, abscesses, rheumatism, syphilis and gonorrhea	[30,33]
	Fruit and root decoction	Postparturition hemorrhage	[44]
	Stem bark decoction of *K. africana* and the leaves of *Irvingia gabonensis*	Spleen infection	
	Powdered fruit mixed with palm oil	Dizziness	
	Leaves and stem bark decoction	Malaria	
	Decoction (stem bark of *K. africana* and leaves of *Cassia occidentalis* and potash)	Gonorrhea and syphilis	
	Mixture of ground *K. africana* young fruit and snails rolled into balls and allowed to dry is eaten with a cup of tea every day	Infertility	
	Bark	Rheumatism, regularizing menstrual flow, epilepsy and dysentery	
	Stem bark paste mixed with palm oil and salt	Expelling retained placenta	
	Beer from fruit extract	Children’s bath for the treatment of measles	[22]
	Stem bark and root decoction	Blood cleansing and pelvic pains during pregnancy	
	*Kigelia* and *Searsia nebulosa* stem bark decoction	Dysmenorrhea	[26]
	*Kigelia africana, Hypoxis hemerocallidea* and *Senecio serratuloides* leaves and roots decoction	Sexually transmitted infections and sores	[23]

**Table 2:** Phytochemicals in *Kigelia africana* (Lam.) Benth. responsible for its pharmacological activity.

Classification	Phytochemicals	Plant Part	Reference
Phenolic Compounds	p-Coumaric acid	Stem bark, fruits, roots	[14,46,47]
	Caffeic acid	Stem bark, fruits, roots	[14,46]
	Ferulic acid	Stem bark, fruits	[11,35,47,48,49,50]
	Atranorin	Stem bark	[51]
	Nonacosanoic acid, 2-(4-hydroxyphenyl) ethyl ester	Stem bark	[14,46]
	Luteolin	Roots, leaves, wood	[12,42]
	Luteolin 7-O-glucoside	Leaves	[35]
	6-hydroxyluteolin	Roots, leaves, wood	[42]
	6-p-coumaroyl-sucrose	Fruits	[52]
	Kigeliol	Wood	[11,47,50,52]
	Balaphonin	Stem bark	[3,6,12,42,53]
Coumarins	Kigelin	Roots, stem bark, leaves, wood	[14]
	8-hydroxy-6, 7-dimethoxy-3-methyl-3, 4-dihydroisocoumarin	Roots	[11,50]
	Isokigelin	Stem bark	[52,53]
	6-Demethylkigelin	Roots, stem bark	[52]
	6-Methoxymellein		
	1,3-dimethylkigelin	Stem bark	[11,12,46]
Sterols	β-Sitosterol	Stem bark, fruits, heartwood, roots	[14,50,53,54]
	Stigmasterol	Stem bark, roots, heartwood	[3,54,55]
	γ-sitosterol	Stem bark, fruits	[3,47]
Triterpenes	Oleanolic acid	Stem bark	[3,14,46,51]
	Pomolic acid		
	2β,3β,19α-Trihydroxy-urs-12-en-28-oic acid		
Diterpenes	Phytol	Leaves	[56]
	3-Hydro-4,8-phytene		
Unsaturated Fatty acids	(9Z,12Z)-Methyl octadeca-9,12-dienoate	Leaves	[56]
	Vernolic acid	Stem bark, roots, leaves, heartwood	[3,12,42]
	Methyl-12-methyltetradecanoate	Leaves	[3,35]
	Palmitic acid or hexadecanoic acid	Leaves, flowers	[52]
Quinones	Lapachol	Stem bark, fruits, roots, heartwood	[14,35,46,48]
	Dehydro α-lapachone	Stem bark, fruits, roots, heartwood	[14,52,53]
	2-acetylfuro-1,4-naphthoquinone	Stem bark	[14,46,48]
	Kigelinol	Stem bark, roots, fruits	[14,46]
	Kigelinone	Stem bark	[49,57,58]
	Isokigelinol	Roots, stem bark, roots, fruits	[3,41]
	Pinnatal	Roots, fruits, stem bark	[47,49,57,58]
	Isopinatal	Roots and fruits, stem bark	[49,54,57]
	Norviburtinal	Root bark, stem bark, fruits	[12,47,59]
	Sonovoburtinal	Root bark	[47]
	2-(1-Hydroxyethyl)-naphtho[2,3-b] furan-4,9-quinone	Roots, stem bark	[58]
	Kigelinone	Root and fruit, stem bark, heartwood	[48,54]
	2-acetylnaphtho[2,3-b] furan-4,9-quinone	Stem bark, roots	[3]
	2-(1-hydroxyethyl) naphtho [2,3-b] furan-4,9-dione	Stem bark, roots	[3]
	Tecomaquinone-I	Heartwood	[60]
	Kojic acid	Stem bark	[46]
Iridoids	7-Hydroxyviteoid II	Fruits	[11,60,61,62]
	7-hydroxy-10-deoxyeucommiol		
	10-Deoxyeucommiol		
	Jiofuran		
	3-(2-hydroxyethyl)-5-(2-hydroxypropyl)-4,5-dihydrofuran-2(3H)-one		
	7-hydroxyeucommic acid		
	7-hydroxy eucommiol	Twig, roots, leaves, wood	[3,62]
	Jioglutolide		
	1-Dehydroxy-3,4-dihydroaucubigenin		
	Des-p-hydroxy benzoyl kisasagenol B		
	Ajugol		
	6-Trans-caffeoyl ajugol		
	Verminoside	Stem bark, fruits, twigs leaves, roots	[12,22,23,28,36,49,61,62,63]
	Specioside	Stem bark	[49,62,64]
	Minecoside	Stem bark	[11]
Alkanes	n-hentriacontane	Leaves	[11]
	11-(2,2-dimethylpropyl) heneicosane		
	2,6,10-trimethyldodecane		
	4,4-dimethylundecane		[3,35]
	1-iodohexadecane		[35]
	1-iododecane		
	Tritriacontane		[62]
	Hentriacontane		
	Nonacosane		
	Heneicosane		[11]
Esters	Pentafluoro-N-heptadecyl	Leaves	[11]
	2-ethylhexyloctadecyl sulfurous acid	Leaves	
	2-(4-hydroxyphenyl) ethyl ester	Bark	[14]
	Ethyl linoleoate	Leaves, flowers	[52]

**Table 3:** Pharmacological activity of *Kigelia africana* extracts.

Activity	Plant Part	Extract	Organism	Observed Effect	Compounds Responsible	Reference
In vitro antibacterial activity	Leaf	Ethanol	*E. coli*	Maximum activity (22 mm)	Specioside, verminoside	[16,42,45]
			*P. vulgaris*	Moderate activity		
			*K. pneumoniae*	Resistant		
			*C. amalonaticus*	Resistant		
		Aqueous	*C. amalonaticus*	Maximum activity (7 mm)		
			*S. aureus*	Moderate activity (5 mm)		
			*P. aeruginosa*	Moderate activity (4 mm)		
			*P. vulgaris*	Moderate activity (4 mm)		
			*K. pneumoniae*	Resistant		
			*C. amalonaticus*	Moderate activity		
			*S. aureus*	Moderate activity		
			*P. aeruginosa*	Moderate activity		
			*P. vulgaris*	Moderate activity		
			*K. pneumoniae*	Least activity		
		n-hexane	*P. vulgaris*	Maximum activity (6 mm)		
			*S. aureus*	Minimum activity (2 mm)		
			*K. pneumoniae*	Resistant		
			*P. aeruginosa*	Resistant		
			*C. amalonaticus*	Resistant		
			*E. coli*	Resistant		
	Fruit	Aqueous	*P. vulgaris*	Maximum activity (6 mm)		
			*C. amalonaticus*	Maximum activity (6 mm)		
			*S. aureus*	Moderate activity		
			*E. coli*	Moderate activity		
			*K. pneumoniae*	Resistant		
			*P. aeruginosa*	Resistant		
			*P. vulgaris*	Maximum activity (23 mm)		
			*C. amalonaticus*	Minimum activity		
	Bark	Ethanol	*E. coli*	Maximum activity (10 mm)		
			*P. aeruginosa*	Moderate activity		
			*P. vulgaris*	Moderate activity		
			*S. aureus*	Least activity (1 mm)		
			*K. pneumoniae*	Resistant		
			*C. amalonaticus*	Resistant		
	Bark	n-hexane	*C. amalonaticus*	Maximum activity (4 mm)		
			*E. coli*	Minimum activity (2 mm)		
			*S. aureus*	Resistant		
			*P. aeruginosa*	Resistant		
			*P. vulgaris*	Resistant		
			*K. pneumoniae*	Resistant		
	Bark	Aqueous	*S. aureus*	Maximum activity (15 mm)		
			*P. aeruginosa*	Moderate activity (5 mm)		
			*P. vulgaris*	Moderate activity (5 mm)		
			*C. amalonaticus*	Minimum activity		
			*K. pneumoniae*	Resistant		
			*E. coli*	Resistant		
			*E. coli*	Very good activity (20 mm)		
			*C. amalonaticus*	Least activity (3 mm)		
Antifungal activity	Fruit	Methanol	*P. chrysogenum*	Resistant		[45]
			*A. niger, C. albicans* and *P. chrysogenum*	Strong inhibition (1238, 841.2 and 989.7 µg/mL, respectively)		
			*C. neoformans*	Resistant		
		Aqueous	*A. niger, C. albicans* and *P. chrysogenum*	Strong inhibition (2487, 2060 and 2768 µg/mL, respectively)		
			*P. chrysogenum*	Resistant		
		Ethyl acetate	*A. niger, C. albicans* and *P. chrysogenum*	Moderate inhibition (1463, 1278 and 1744 µg/mL, respectively)		
			*P. chrysogenum*	Resistant		
Anti-inflammatory and Analgesic Activity	Leaf	Methanol	Mice	First hour at the dose of 150 mg/kg and 15 mg/kg body weight for reference drug diclofenac reduced hind paw diameter by 0.21% and 1.10%, respectively	Verminoside	[45,60,74,75,76,77]
				Second hour at the dose of 100 mg/kg and 150 mg/kg body weight for reference drug diclofenac reduced hind paw diameter by 0.42% and 1.42%, respectively		
				Third hour, the extract at the dose levels of 50 mg/kg, 100 mg/kg and 150 mg/kg body weight, as well as diclofenac, reduced the inflamed hind paw diameter by 0.86%, 2.25%, 3.41% and 4.02%, respectively		
				Fourth hour at the doses of 50, 100 and 150 mg/kg body weight reduced inflamed hind paw diameter by 1.95%, 2.98% and 4.98% and reference drug reduced inflamed paw diameter by 4.43%		
	Leaf	Methanol	Wistar rats	Higher dose (400 mg/kg) exhibited better analgesic activity than the lower dose (200 mg/kg)		
Antidiabetic Activity	Leaf	Aqueous and ethyl acetate	Swiss albino mice	Extracts showed a blood glucose-lowering effect		[42,79]
Antiprotozoal Activity	Root bark	Hexane, dichloromethane and ethyl acetate	*P. falciparum*, *T. brucei* and *T. brucei rhodesiense*	Significant anti-plasmodial activity	Specioside, 2β, 3β, 19α-trihydroxyurs-12-en-28-oic acid, atranorin, p-hydroxycinnamic acid, Lapachol, verminoside and minecoside	[48,61]
	Stem bark	Butanol	*E. histolytica*	Significant antiplasmodial activity	2-(1-hydroxyethyl)- naphtho-[2,3-b]-furan-4,9-quinone, isopinnatal, kigelinol and isokigelinol	[58]
Antiurolithiatic Activity	Fruit	Ethanol	Male wistar albino rats	Reduced the elevated urinary oxalate, uric acid and phosphate		[94]
Anticonvulsant Activity	Bark	Aqueous and Methanol	Wistar rats	Potent anticonvulsant activity	Linoleic and cinnamic acid	[95]
Antidiarrheal Activity	Bark	Ethanol	Mice	Significantly inhibited the castor oil-induced diarrhea in mice, with 500 mg/kg for extract and atropine (positive control) 62.7% and 82% P, respectively.		[96]
Treatment of Sexually Transmitted Diseases	Roots	Aqueous	*C. albicans*	strong activity, even in the absence of emulsin	Iridoids	[96,97]
Antidiuretic Activity	Bark	Aqueous	Male wistar albino rats	Strong diuretic activity		[98,99]
Antioxidant Activity	Bark, Fruit and Leaf	Methanol		Quercetin (positive control)—94% inhibition, bark—(67.33%) inhibition, fruit—(62.66%) inhibition and leaves—(59.66%) inhibition	Phenols	[16,102]
	Root	Ethyl acetate		High antioxidant activity		[33,103]
	Fruit	Methanol		Significantly reduced the production of thiobarbituric acid reactive substances (TBARS)		[104]
Anticancer Activity	Stem bark	Methanol		Cytotoxic against human tumor cell lines with IC50 values of 15.1 ± 3.4, 126.0 ± 9.1, 90.7 ± 4.7, 186.0 ± 9.2, 101.0 ± 7.4, 124.1 ± 8.9, 11.8 ± 3.8 and 10.2± 2.7 µg/mL against T-cell leukemia, acute lymphoid leukemia, acute myeloid leukemia, chronic myeloid leukemia, non-Hodgkin’s lymphoma, Hodgkin’s lymphoma, breast cancer and murine lung cancer cell line, respectively	Dimethylkigelin, kigelin, ferulic acid and 2-(1-hydroxyethyl)-naphtho[2,3-b] furan-4,9-dione	[108,109]
	Leaf	Methanol	Mice	Decrease in tumor size		[111]
	Fruit			Potent against melanoma and breast cancer cell lines		[112]
Toxicity	Fruit	Methanol	Male Sprague–Dawley rats	LD50 Extract was safe (LD50 was 3981.07 mg/kg) 3981.07 mg/kg		[38,51]
	Polyherbal	Aqueous	Male Wistar albino rats	After the 30 days, there was no effect on hematological, urinary and plasma biochemical parameters		[117]
